# Extra Protection for Pregnant Women: Calcium Supplement Reduces Blood Lead

**Published:** 2009-01

**Authors:** Carol Potera

Lead, like calcium, is stored in bones and generally does not circulate throughout the body. But the demands of pregnancy and lactation trigger the release of calcium, which also releases lead into the maternal blood stream. Researchers previously showed that daily calcium supplementation during lactation reduced maternal blood lead by 15–20% and lead in breast milk by 5–10%. A new study by the same team shows that taking inexpensive calcium supplements daily also reduces blood lead levels during pregnancy **[*EHP* 117:26–31; Ettinger et al.]**. Such supplementation could help mitigate the adverse effects of prenatal lead exposure, which include low birth weight, lower intelligence scores, and impaired motor and visual skills.

The study included 557 women recruited in the first trimester of pregnancy from prenatal clinics in Mexico City. The women were recruited from 2001 to 2003; Mexico completed the phase-out of leaded gasoline in 1997, so women enrolled in the study had been exposed for many years to high environmental lead levels prior to becoming pregnant. In addition, just over one-third of the women used the traditional lead-glazed pottery that is common in Mexico. Half the women received 1,200 mg of calcium daily and the others received placebos.

Blood lead levels were checked in the first (baseline), second, and third trimesters of pregnancy. The Mexican women enrolled in the current study had an estimated average dietary calcium intake of 900 mg per day, which parallels national surveys of U.S. women. (The U.S. Institute of Medicine advises 1,000 mg of calcium daily for pregnant and lactating women aged 19–50 years and 1,300 mg/day for pregnant and lactating women under age 19 years.)

Blood lead levels declined more in the second trimester than in the third, with reductions averaging 14% and 8%, respectively. Women who were more compliant with the calcium regimen had higher reductions in blood lead relative to the placebo group. The most compliant women—those who took at least 75% of their calcium supplements—showed a 24% drop in blood lead levels over the course of pregnancy, with the greatest reduction (31%) occurring in women who were most compliant and who also cooked, served, or stored food in lead-glazed pottery, and who had the highest bone lead levels. The investigators conclude that calcium supplements should be considered as a low-risk, cost-effective means for lowering fetal lead exposure.

## Figures and Tables

**Figure:**
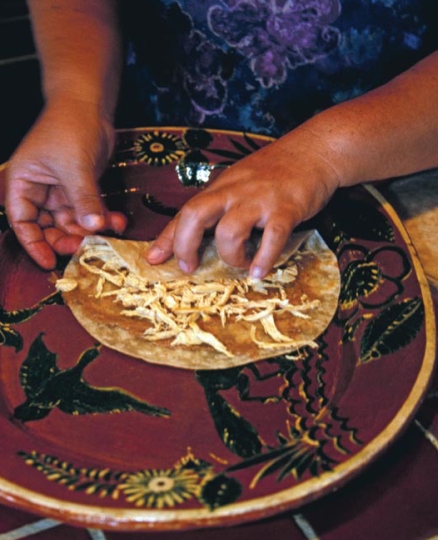
The greatest reduction in blood lead was seen in women who were most compliant with the calcium regimen and who used lead-glazed pottery.

